# Age-dependent regulation of obesity and Alzheimer-related outcomes by hormone therapy in female 3xTg-AD mice

**DOI:** 10.1371/journal.pone.0178490

**Published:** 2017-06-02

**Authors:** Amy Christensen, Christian J. Pike

**Affiliations:** Leonard Davis School of Gerontology, University of Southern California, Los Angeles, California, United States of America; Hospital Infantil Universitario Nino Jesus, SPAIN

## Abstract

Depletion of ovarian hormones at menopause is associated with increased Alzheimer’s disease (AD) risk. Hormone loss also increases central adiposity, which promotes AD development. One strategy to improve health outcomes in postmenopausal women is estrogen-based hormone therapy (HT), though its efficacy is controversial. The window of opportunity hypothesis posits that HT is beneficial only if initiated near the onset of menopause. Here, we tested this hypothesis by assessing the efficacy of HT against diet-induced obesity and AD-related pathology in female 3xTg-AD mice at early versus late middle-age. HT protected against obesity and reduced β-amyloid burden only at early middle-age. One mechanism that contributes to AD pathogenesis is microglial activation, which is increased by obesity and reduced by estrogens. In parallel to its effects on β-amyloid accumulation, we observed that HT reduced morphological evidence of microglial activation in early but not late middle-age. These findings suggest that HT may be effective during human perimenopause in reducing indices of obesity and AD-related pathology, a conclusion consistent with the window of opportunity hypothesis.

## Introduction

Alzheimer’s disease (AD) is a multifactorial, age-related neurodegenerative disorder. Modifiable AD risk factors include obesity and its associated disorders metabolic syndrome and type 2 diabetes [1], which are most strongly linked to disease promotion at middle-age [2]. Another midlife AD risk factor is estrogen depletion that occurs in women around menopause [3, 4]. The depletion of ovarian hormones at menopause is associated with significant systemic changes, including slowed metabolism and increased central adiposity [5, 6]. Mechanistically, central adiposity may contribute to several diseases by increasing systemic and neural inflammation [7], which are established risk factors for the initiation and progression of AD [8, 9]. Because estrogens protect against both the development [10] and deleterious effects [11] of obesity, estrogen-based hormone therapy (HT) may offer an effective intervention for the prevention of obesity and its associated disorders. Unknown is whether and how obesity and estrogens may interact to affect (i) the development of AD pathology, and (ii) the established ability of estrogens to attenuate AD pathology.

Although numerous studies have linked HT use in women with decreased risk of AD [12–17], a large clinical trial found that HT use at late menopause increased rather than decreased dementia risk [18]. Subsequent work suggests that the protective effects of HT against AD may require initiation of treatment near the onset of menopause as opposed to several years after menopause [13, 19–21], when the brain may exhibit diminished estrogen responsiveness and/or AD pathogenesis is too advanced to effectively prevent.

Neither the possibility that obesity and estrogen interact in the regulation of AD nor the hypothesis that the efficacy of estrogen treatment depends upon the age of treatment initiation has been rigorously investigated. Here, we studied the effects of obesity in the presence and absence of estrogen-based HT on metabolic and AD-related outcomes in female 3xTg-AD mice during both early (7–9 months) and late (16 months) middle-age. These chronological ages are associated with reproductive changes in female rodents that model key aspects of human perimenopause and menopause [22, 23], thus permitting insights into the interactions between obesity, ovarian hormones, and AD and how these relationships are modulated by reproductive aging. Our findings indicate that obesity accelerates the initial development of AD-related pathology, but not its continued progression. Further, HT was observed to protect against metabolic effects of obesity more strongly in early than late middle-age. AD-related pathology was reduced by HT only in early middle-age. These findings have important implications for further investigation into HT administration at perimenopause to women at risk for AD.

## Materials and methods

### Animals

Female 3xTg-AD mice were maintained in a colony at the University of Southern California under controlled temperature and a 12:12 light/dark schedule (lights on at 0600) and had *ad libitum* access to food and water. Mice were singly housed upon reaching the required age range for study enrollment and were euthanized by carbon dioxide inhalation followed by cervical dislocation at termination of the experimental period. Mice were monitored daily for general health, appearance, and criteria for euthanasia (e.g., >10% body weight, lethargy and poor grooming); no mice were prematurely euthanized. Three different ages of mice were studied: young (23–25 days), early middle-age (7–9 mo), late middle-age (16–17 mo) at the initiation of the four-month experimental period. Two experimental conditions of young mice were enrolled with n = 6 for normal fat diet and n = 7 for high-fat diet. There were no deaths in the young experimental groups. Early middle-aged mice in four experimental groups were enrolled with n = 8 per group. To confirm onset of reproductive aging in these mice, ovulatory cyclicity was determined by cytology of daily vaginal smears over the 14 days prior to enrollment, as previously described [23]. Mice were randomized to treatment groups while also ensuring that all treatment groups included the same number of animals from each cycle status: 2 regular cyclers (normal 4–5 day cycles), 4 irregular cyclers (two consecutive cycles >5 days), and 2 acyclic animals (no cycles, mostly in constant estrus). In the early middle-age mice, there were a total of six deaths across the eight groups before completion of the experiment, yielding the following numbers of mice per group: normal fat diet + vehicle = 7, normal fat diet + hormone therapy (HT) = 5, HFD + vehicle = 8, HFD + HT = 6. Consistent with prior observations in aging female mice [24, 25], we find that nearly all 3xTg-AD are acyclic at late middle-age. In the late middle-age mice, there was a greater than expected loss of animals across all treatment groups, especially in the HT-treated groups. The observed deaths at this age are not inconsistent with reports of increased frailty and mortality in 3xTg-AD mice [26]. All late middle-age animals were treated with oral antibiotics for the final 12 days of the study (after the final capsule implant) to increase likelihood of survival to the predetermined euthanasia date. Fourteen late middle-age mice were enrolled in the two vehicle control groups, of which 12 completed the study. The two HT groups were enrolled with a total of 22 animals, but only 10 animals survived to the completion of the experiment. The cause of death was not definitively determined, but a majority of the deceased mice exhibited pyometra upon necropsy. The number of mice in the late middle-aged groups that completed the study and were analyzed are: normal fat diet + vehicle = 6, normal fat diet + HT = 4, HFD + vehicle = 6, HFD + HT = 6.

All procedures were conducted in accordance with National Institutes of Health guidelines, under the supervision of veterinary staff, and following a protocol (#11717) approved by the University of Southern California Institutional Animal Care and Use Committee.

### Hormone therapy

Mice in the early and late middle-age groups received either vehicle or HT consisting of continuous 17β-estradiol (E2) and discontinuous (10 days during each of four consecutive 31-day periods) progesterone (P4), as previously described [27, 28]. Animals were anesthetized with 2–3% isoflurane then implanted subcutaneously (between the shoulder blades) with a Silastic capsule (1.47 mm ID x 1.96 mm OD; Dow Corning, Midland, MI). Each capsule had a total length of 9 mm with the inner 5 mm packed with cholesterol (vehicle) or a 1:1 mixture of E2 and cholesterol, consistent with a previously characterized design [29]. After three weeks, animals were implanted with a new E2 or vehicle implant; capsules were then replaced every 31 days for the remainder of the 4-month experimental period. Estradiol-treated animals also received a slow-release subcutaneous P4 pellet (2.8 mg, 10 day release; Innovative Research of America, Sarasota, FL); a new P4 pellet was added with the estradiol capsule for all four months.

### Diet-induced obesity

Animals were maintained on a standard high-fat (HF; D12492) or control, normal fat (NF; D12450B) diet purchased from Research Diets, Inc. (New Brunswick, NJ). These are defined diets that differ primarily in the presence or absence of lard. The HF diet contains 60% (kcal) fat with 20% protein and 20% carbohydrate, whereas the NF diet is 10% fat, 20% protein, and 70% carbohydrate. These are nutrient-matched diets that utilize the same ingredients with the exception of corn starch added to the NF diet to help offset the increased energy of the lard. Body weight and food intake were monitored weekly.

### Glucose measurements

Fasting glucose measurements were made at the initiation of experimentation and monthly thereafter. Following overnight fasting (~16 hours), blood was collected by piercing the lateral tail vein. Twenty microliters of blood was collected on a glucose test strip and assayed using a Precision Xtra glucose monitor (Abbott Laboratories, Abbott Park, IL). In young females, the initial fasting glucose was not measured. Instead, a glucose tolerance test was performed at the end of the treatment. In brief, animals were oral gavaged with 2g/kg D-glucose. Blood glucose levels were measured at 0, 15, 30, 60 and 120 minutes following glucose administration.

### Spontaneous alternation behavior

Four to five days after the final E2 capsule and P4 pellet implant, animals were tested for spontaneous alternation behavior in a Y-maze. Animals were allowed to acclimate to the behavior room for 30 minutes prior to testing. Animals were placed in the long arm of a Y-maze facing away from the other arms to start the test. Arm entries (at least two paws into an arm) were recorded for 3 minutes. Animals with fewer than 10 or more than 35 entries were excluded from analysis; the lower limit was relaxed for the late middle-aged animals.

### Tissue collection

At the end of the four-month treatment period, animals were euthanized and the brain was rapidly removed and hemisected. Half of the brain was immersion fixed in 4% paraformaldehyde in 0.1M Sorenson’s phosphate buffer for 48 hours at 4°C. From the other half, the hippocampus was dissected and immediately frozen at -80°C for subsequent RNA extractions. The right retroperitoneal fat pad and right visceral (which included gonadal and uterine fat) fat pad were dissected and weighed. Blood was collected from each animal. Plasma was separated by centrifugation and stored in aliquots at -80°C until assayed.

### Plasma assays

Triglycerides were assayed using a LabAssay triglyceride kit (Wako, Irvine, CA). Cholesterol levels were measured using a total cholesterol colorimetric kit (BioVision, Milpitas, CA). Estradiol was determined using by ELISA kit (Cayman, Ann Arbor, MI). All kits were used according to the manufacturers’ instructions. Progesterone levels were not assayed because euthanizing occurred following their transient exposure period. Efficacy of discontinuous progesterone in this HT paradigm has been previously demonstrated [27].

### Immunohistochemistry

Fixed hemibrains were sectioned exhaustively in the horizontal plane at 40 μm using a vibratome (Leica Biosystems, Buffalo Grove, IL). Sections were stored singly in PBS with 0.03% sodium azide at 4°C until immunohistochemistry was performed. Every eighth section (from a total of ~100) was immunostained for β-amyloid (Aβ) as previously described [30]. In brief, tissue sections containing hippocampus were pretreated with 95% formic acid for 5 min, then washed 3 times for 5 min in TBS, followed by a 10 min rinse with an endogenous peroxidase blocking solution. Next, sections were rinsed in TBS/0.1% Triton-X before being blocked for 30 min in TBS with 2% BSA. Sections were incubated overnight at 4°C with anti-Aβ antibody (1:300; Life Technologies, Grand Island, NY; Cat #71–5800) diluted in the block solution. Immunostaining was also conducted in the absence of formic acid pretreatment with the following primary antibodies: phospho-PHF-Tau, clone AT8 (1:750; Thermo, Waltham, MA) for phosphorylated tau (phospho-tau), Iba-1 (1:2000; Wako, Irvine, CA) for microglia, and amyloid precursor protein, C-terminal (751–770) (1:16,000; Millipore, Billerica, MA) for C-terminal fragments of amyloid precursor protein. Sections incubated in primary antibody were washed and incubated with the appropriate secondary antibody (Vector Laboratories, Burlingame, CA) for 1 hour and processed for diaminobenzidene staining using Vectastain ABC Elite kit (Vector). Stained sections were air-dried overnight, dehydrated, then coverslipped with Krystalon (EMD Millipore, Billerica, MA).

### Immunohistochemical quantification

Aβ burden was determined as previously described [30, 31]. In brief, sections immunostained for Aβ were visualized with an Olympus BX40 equipped with an OLY-105 camera, allowing for images to be digitally captured then analyzed using NIH Scion Image v1.62. Grayscale images were converted to a binary black/white image using a constant threshold for determination of the percent area of staining (Aβ load). Load values were determined from 2–3 non-overlapping fields of both subiculum and CA1 hippocampus of each section (6–8 sections per animal). Load values were averaged from each section then across all sections in each animal to calculate load values.

Activation state of microglia was based on morphological analysis of Iba-1 staining in a manner consistent with prior reports [32, 33]. Numbers of Iba-1 immunoreactive cells in the hippocampus were estimated by two-dimensional cell counts using random-sampling based on the optical dissector technique, which has been previously used to estimate the number of total cells in the hippocampus [34, 35]. Briefly, an Olympus BX50 microscope equipped with a motorized stage and computer-guided CASTGrid software (Olympus, Denmark) was used for unbiased sampling. In every eighth section containing well-defined CA1-CA3 subregions of hippocampus (6–8 sections per brain), the hippocampus (excluding the dentate gyrus) was outlined from which high magnification microscopic fields were randomly sampled with an X-Y step of 150 μm x 150 μm. Within each field, cells within a counting frame (476 μm^2^) were used for analysis. Microglia were classified as either type 1, (many thin, ramified processes), type 2 (short, thick processes and a rod-shaped cell body), or type 3 (no or few short non-ramified processes or many filapodial processes) cells. The number of cells quantified per brain ranged from 165 (young) to 960 (aged).

Tau-positive cells were counted from 6–8 sections per brain. Only cells that were darkly stained across the majority of the soma were scored. Because blood vessels in the brain also show non-specific staining for Tau, all objects counted were required to be cell-shaped with rounded edges and the approximate size of a cell body.

### PCR

RNA extractions and real-time PCR were performed as previously described [36]. Dissected hippocampi were lysed using TRIzol reagent (Life Technologies; Carlsbad, CA) and processed for RNA extraction using a PureLink RNA Mini Kit (Life Technologies). Purified RNA (1 μg) was used for reverse transcription using the iScript synthesis system (Bio-Rad; Hercules, CA). The resulting cDNA was used for real-time quantitative PCR using a Bio-Rad CFX96 Touch Real-Time PCR Detection System. The amplification efficiency was estimated from the standard curve for each gene. Relative quantification of mRNA levels from various treated animal hippocampi was determined by the ΔΔCt method [37]. The following primer pairs were used: TSPO: forward, 5′-CAGTGTCCTTCACGGAACAA-3′; reverse, 5′TGAATACAGTGTGCCCCAGA-3′; CD11b: forward, 5′-CCAAGACGATCTCAGCATCA-3′; reverse, 5′-TTCTGGCTTGCTGAATCCTT-3′; CD33: forward; 5′ACAGGCTCATCAGCAGGACT-3′; reverse, 5′AGAGCAAAGCTTGGTGCATT-3′; IL-1β: forward, 5′-GGGCCTCAAAGGAAAGAATC-3′; reverse, 5′-TACCAGTTGGGGAACTCTGC-3′; TNFα: forward, 5′-CGTCAGCCGATTTGCTATCT; reverse, 5′-CGGACTCCGCAAAGTCTAAG-3′; IL-6: forward, 5’-AGTTGCCTTCTTGGGACTGA-3’; reverse, 5’-TCCACGATTTCCCAGAGAAC-3’. β-actin: forward, 5′AGCCATGTACGTAGCCATCC-3′; reverse, 5′CTCTCAGCTGTGGTGGTGAA-3′.

### Statistics

All data are reported as the mean ± the standard error of the mean. Data were analyzed using GraphPad Prism version 5. Two-way repeated measure analysis of variance followed by Bonferoni post tests were run for all data measured over time. Two-tailed t-tests were run on the young animals, which had only two groups for analysis. In the early and late middle-age groups, two-way analysis of variance was used followed by Newman-Keuls test when appropriate. Comparisons with *p*<0.05 were considered significant.

## Results

### High-fat diet worsened metabolic indices and AD neuropathology in young mice

**Fig 1:**
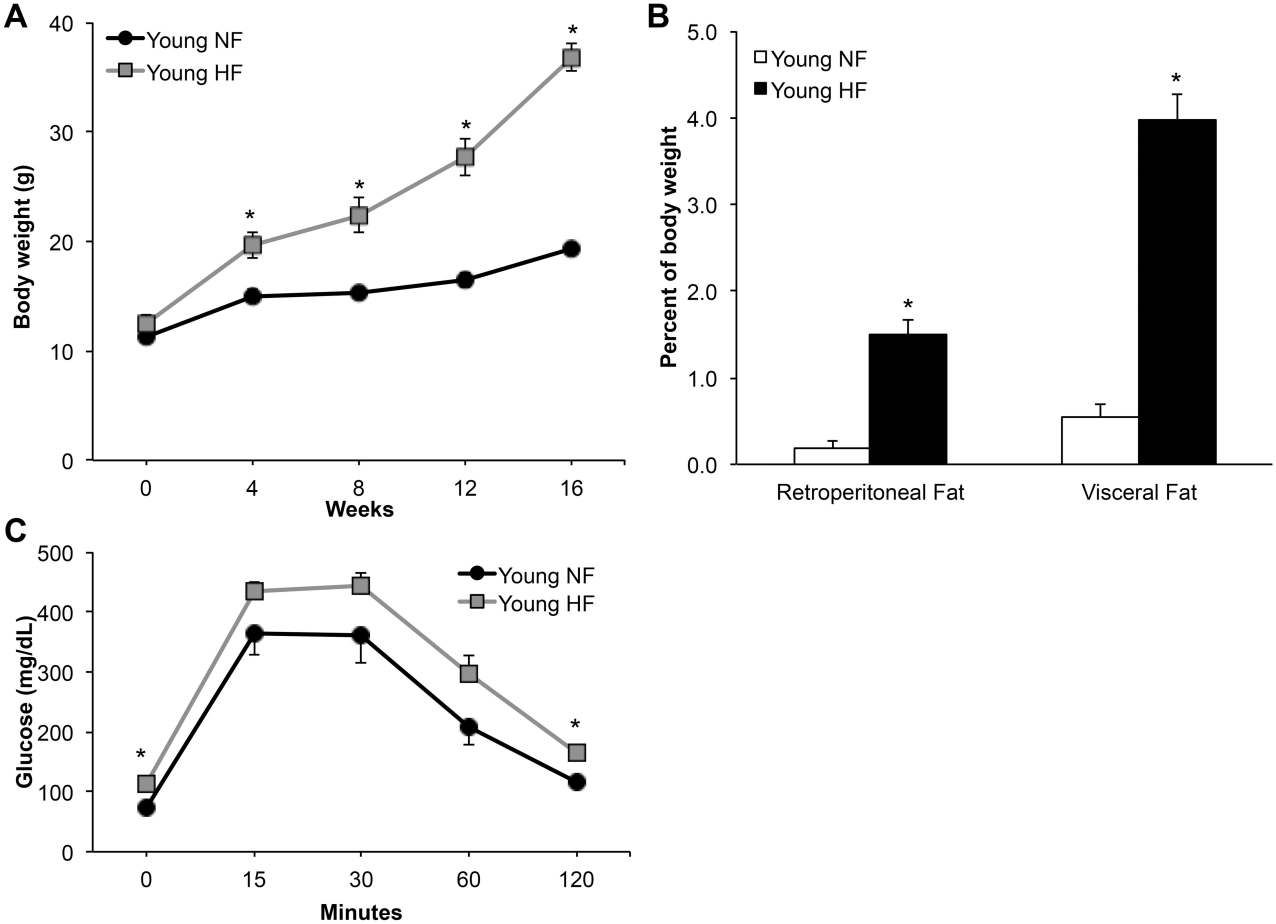
Young female 3xTg-AD mice are obese and show metabolic dysfunction in response to a high-fat diet. (A) Within 4 weeks, young females fed a high-fat (HF) diet (n = 7) were significantly larger than their normal fat (NF) diet (n = 6) fed counterparts. This difference was maintained for the next 12 weeks. (B) After 16 weeks on diet, relative fat pad weights in the HF diet group were significantly larger than those in the NF group. (C) Young females on HF diet showed impaired performance on a glucose tolerance test. In (A) and (C) black lines indicate Young NF, gray lines indicate Young HF. In (B) white bars indicate Young NF and black indicate Young HF. Data show mean ± SEM. Asterisks denote statistical significance: * p<0.05 relative to Young NF group.

**Fig 2:**
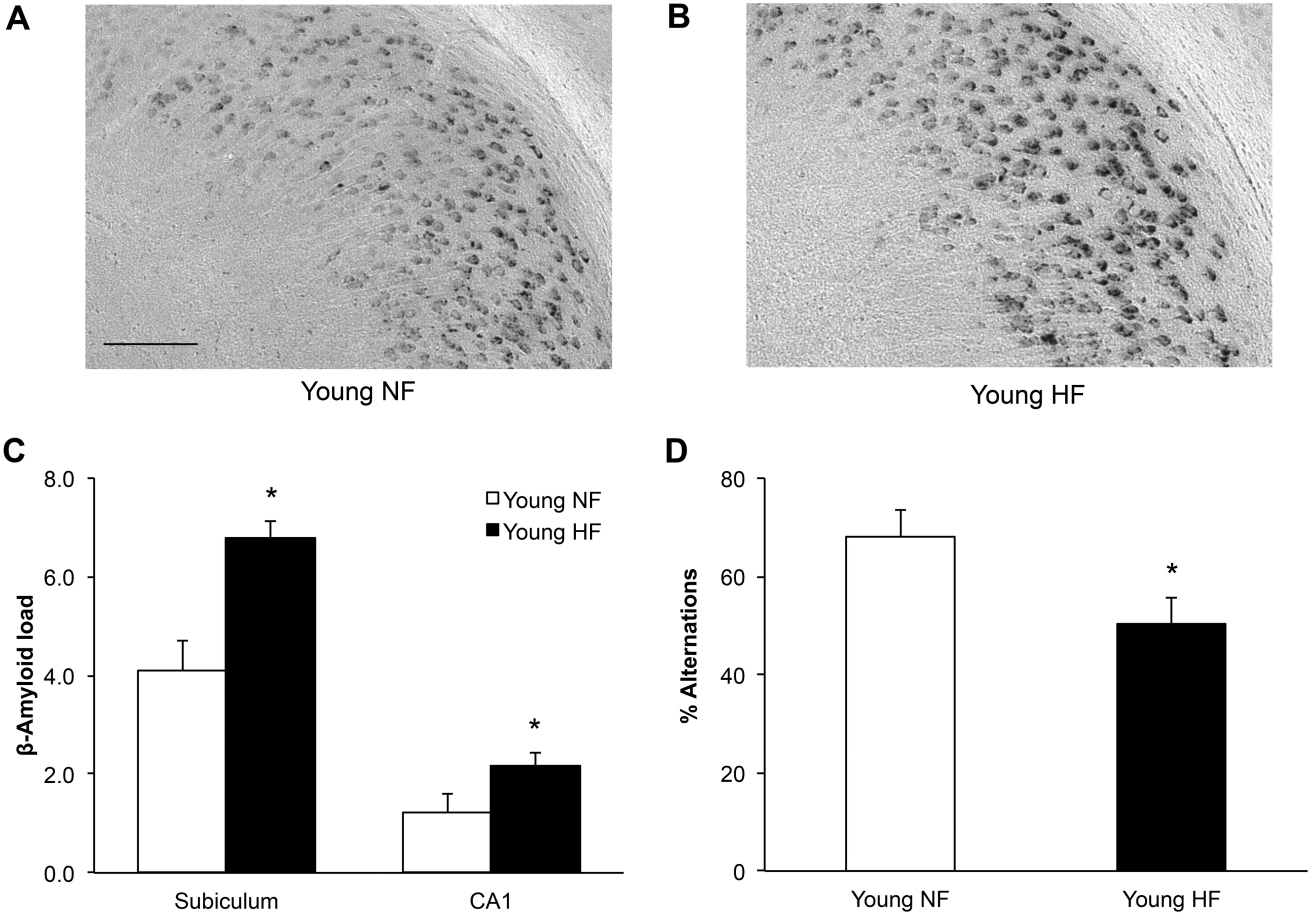
Obese young females show increased hippocampal β-amyloid burden and decreased cognitive performance. Representative images of β-amyloid immunoreactivity from the subiculum of (A) a young NF-fed female and (B) a young HF-fed female illustrate diet-associated effects on β-amyloid burden. (C) HF diet animals showed greater β-amyloid immunoreactive load than those on NF diet. (D) Likewise, HF diet animals show decreased spontaneous alternation in the Y-maze, a test of working memory. White bars denote Young NF animals (n = 6), black denotes Young HF animals (n = 7). Data show mean ± SEM. Asterisks denote statistical significance: * p<0.05 relative to Young NF group.

Previous work has shown that diet-induced obesity in young adult 3xTg-AD mice increases AD-like neuropathology [38]. In order to confirm these results, young female 3xTg-AD mice were fed a normal or a high-fat diet (HFD) beginning soon after weaning (25–30 days old). Over four months, HFD fed animals gained significantly more body weight (Fig 1A; 2-way repeated measures ANOVA; F_diet_(1,11) = 40.8, p<0.0001) and had heavier fat pads (Fig 1B; t-test; retroperitoneal, t = 7.48, df = 11 p<0.0001; visceral, t = 10.9, df = 11 p<0.0001) than mice maintained on normal diet. Additionally, HFD mice showed impaired clearance of glucose during a glucose tolerance test (Fig 1C; 2-way repeated measures ANOVA; F_diet_(1,10) = 6.62; p<0.05). The HFD animals also had significantly higher fasting glucose levels at baseline, prior to the start of the test (72.7 ± 4.3 vs 113.3 ± 10.1 mg/dL; t-test; t = 3.70, df = 10 p<0.005). Plasma levels of triglycerides and cholesterol showed non-significant trends of increased values (Table 1). To evaluate the effects of HFD on AD-related outcomes, young 3xTg-AD mice were examined for both accumulation of Aβ and performance on spontaneous alternation behavior (SAB), a hippocampal-dependent test of working memory and attention. The young HFD animals exhibited significantly greater Aβ burden in both the subiculum and CA1 region of the hippocampus (Fig 2A–2C; t-test; subiculum, t = 4.14, df = 5 p<0.05; CA1, t = 2.03, df = 5, p<0.05) and performed significantly more poorly at SAB (Fig 2D; t-test; t = 2.33, df = 8, p<0.05). Consistent with previously findings [30], Aβ accumulation in young 3xTg-AD mice was largely intra-neuronal and distinct from amyloid precursor protein C-terminal fragments, which in contrast to Aβ do not exhibit increased accumulation with age (S1 Fig). Phospho-tau levels were not measured in young mice as this pathology has yet to develop in 3xTg-AD mice at this age.

**Table 1:** Plasma levels of triglycerides and cholesterol across treatment groups.

Plasma	Young		Early MA		Late MA		Aging p values
	p value	Concentration	p value	Concentration	p values	Concentration	
Triglycerides (mg/dL)	Diet = 0.21	NF = 70.4 ± 6.8	Diet = 0.33	NF + V = 63.3 ± 7.2	**Diet = 0.02**	NF + V = 103.6 ± 23.9	0.13
				NF + HT = 58.5 ± 3.4		NF + HT = 75.6 ± 7.7	
		HF = 86.8 ± 10.6	HT = 0.42	HF + V = 73.5 ± 7.9	HT = 0.11	HF + V = 62.0 ± 7.2	
				HF + HT = 64.9 ± 9.7		HF + HT = 41.0 ± 4.5	
Cholesterol (mg/dL)	Diet = 0.13	NF = 75.2 ± 6.6	**Diet = 0.0002**	NF + V = 90.0 ± 10.1	**Diet = 0.003**	NF + V = 92.2 ± 7.9	0.32
				NF + HT = 110.1 ± 3.4		NF + HT = 89.2 ± 8.5	
		HF = 95.2 ± 10.6	**HT = 0.01**	HF + V = 126.5 ± 6.4	HT = 0.78	HF + V = 135.4 ± 14.7	
				HF + HT = 152.8 ± 8.4		HF + HT = 131.1 ± 14.0	

### Effects of hormone therapy on metabolic and AD-related outcomes in early middle-age mice

**Fig 3:**
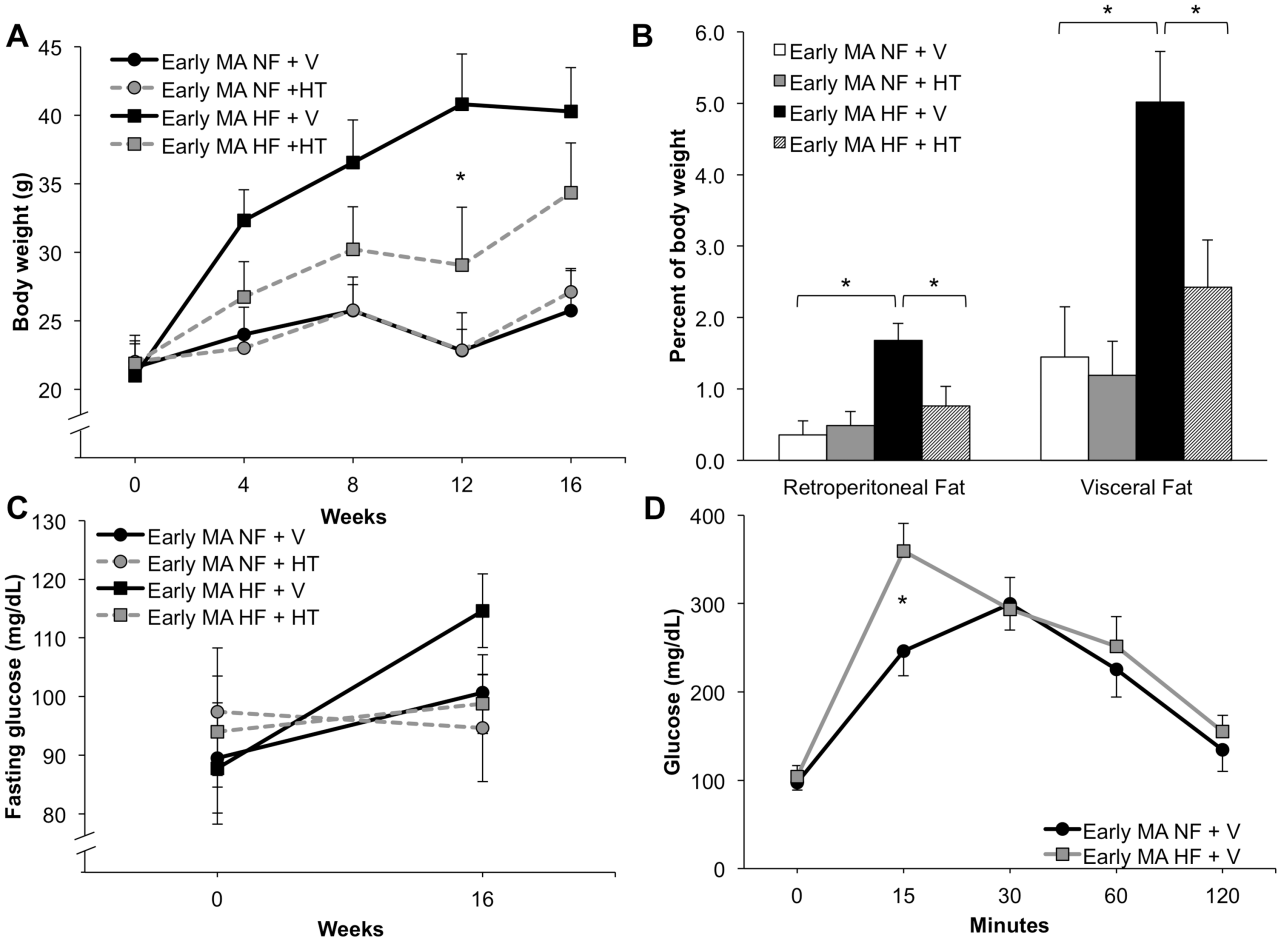
Hormone therapy protected early middle-aged (7–9 months old) mice from the metabolic effects of HF diet. (A) Early middle-aged (MA) female mice on HF diet given hormone therapy (HT) gained less weight than animals treated with vehicle (V). HT had no effect on body weight in NF-fed animals. (B) HF diet increased relative fat pad mass, but HT was able to attenuate this effect. (C) HT showed a non-significant trend of reducing the fasting glucose levels across both diets. (D) Early middle-aged females on HF diet showed a slightly impaired performance on the glucose tolerance test that was significant at the 15 minute time point. In (A) and (C) black circles with a solid line denote NF + V animals, grey circles with a dashed line denotes NF + HT, black squares with a solid line denote HF + V animals, and grey squares with a dashed line denotes HF + HT. In (B) white bar indicates NF + V, grey indicates NF + HT, black indicates HF + V, and striped indicates HF + HT. In (D) NF + V is black and HF + V is grey. Data show mean ± SEM (NF + V = 7, NF + HT = 5, HF + V = 8, HF + HT = 6). Asterisks denote statistical significance: * p<0.05 relative to HF + V in (A), indicated groups in (B), and NF + V in (D).

Female 3xTg-AD mice aged 7–9 months were assigned to one of four groups: normal fat diet + vehicle, normal fat diet + hormone therapy (HT), HFD + vehicle, or HFD + HT. Animals were treated for four months and then euthanized. HT yielded supra-physiological estradiol levels (350±40 pg/ml) compared to vehicle groups, which had estradiol levels below assay sensitivity. HT significantly attenuated the weight gain induced by HFD, but did not alter body weight in mice maintained on normal diet (Fig 3A; two-way repeated measures ANOVA; F(12,96) = 8.47; p<0.0001; Bonferoni test: NF + V vs HF + V at 8, 12 and 16 weeks, p<0.05; HF + V vs HF + HT at 12 weeks, p<0.001). In parallel, retroperitoneal and visceral fat pad weights were also significantly decreased by HT in animals in the HFD but not the normal diet condition (Fig 3B; two-way ANOVA; retroperitoneal, F(1,22) = 4.89, p = 0.04; Newman-Keuls test: NF + V vs HF + V, p<0.05; HF + V vs HF + HT, p<0.05; visceral, F_diet_(1,22) = 4.24, p<0.002; Newman-Keuls test: NF + V vs HF + V, p<0.05; HF + V vs HF + HT, p<0.05). Plasma levels of cholesterol but not triglycerides were significantly increased by both HFD and HT (Table 1).

Fasting glucose levels did not significantly change in any group across the four-month experimental period (two-way repeated measure ANOVA; F(3,25) = 0.15, p = 0.93), although there was a non-significant trend towards increased levels in the HF-treated group (Fig 3C). Glucose tolerance was similar in the vehicle-treated groups, with a modestly poorer outcome in the HFD animals that was statistically significant only at the 15 minute time point (Fig 3D; two-way repeated measures ANOVA; F_diet_(1,12) = 1.45; p = 0.25; Bonferoni test: NF vs HF at 15 min, p<0.05). Long-term progesterone treatment is known to significantly alter glucose metabolism in rodents [39–41], thereby preventing meaningful analysis of glucose tolerance in HT-treated groups.

**Fig 4:**
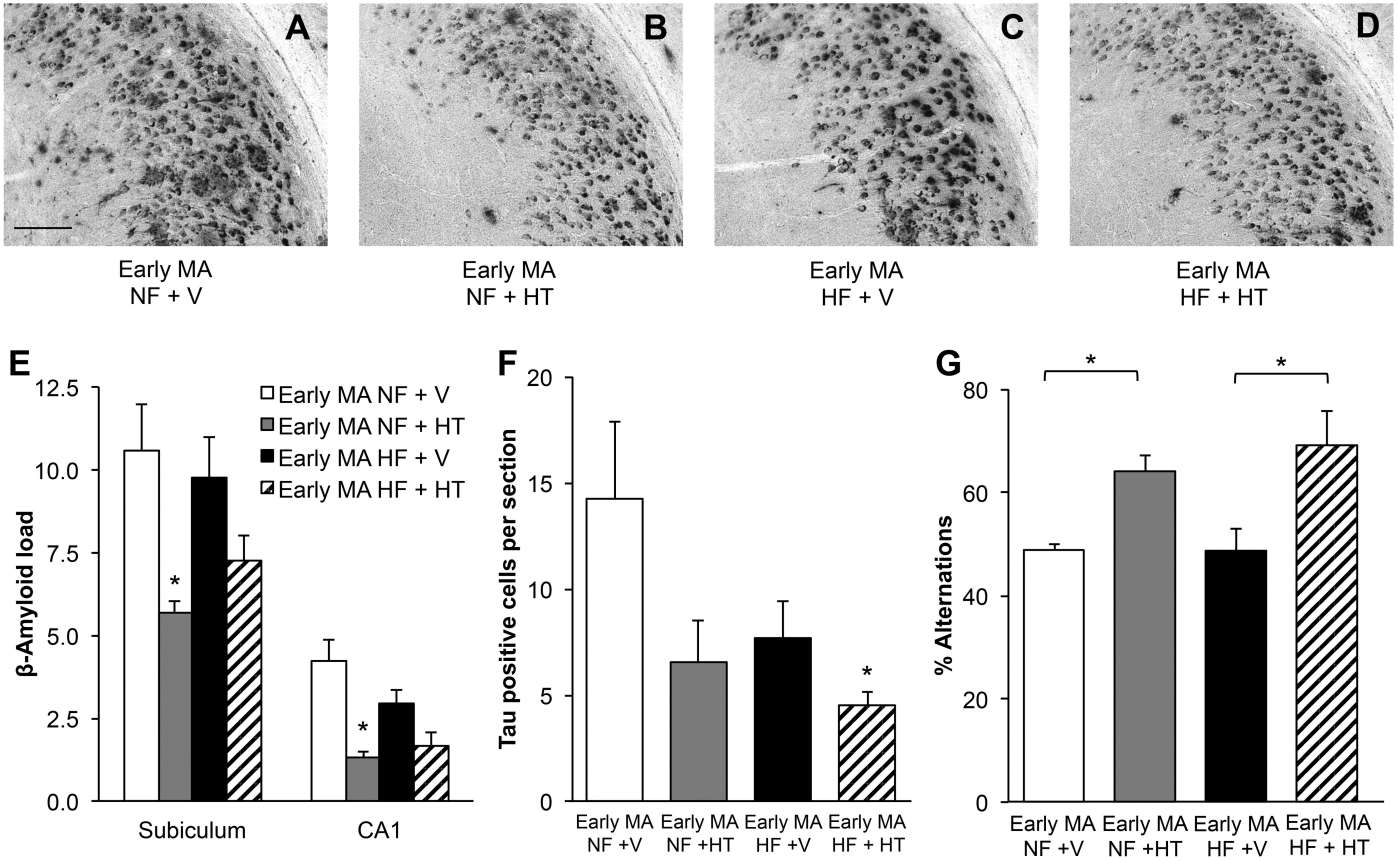
Hormone therapy decreased β-amyloid load and improved cognitive performance in early middle-aged mice. Representative images of β-amyloid immunoreactivity in the subiculum of early middle-aged (MA) mice illustrate the effects of HF diet and HT on β-amyloid load: (A) early middle-aged NF + V, (B) early middle-aged NF + HT, (C) early middle-aged HF + V, and (D) early middle-aged HF + HT. (E) Quantification of β-amyloid immunoreactivity showed that HT was effective in reducing β-amyloid load. (F) The number of cells strongly immunostained with phospho-tau antibody AT8 was not significantly affected by diet but was reduced in the HF group by HT. (G) Performance in the spontaneous alternation test was improved by HT. In (E-G), white bar indicates NF + V, grey indicates NF + HT, black indicates HF + V, and striped indicates HF + HT. Data show mean ± SEM (NF + V = 7, NF + HT = 5, HF + V = 8, HF + HT = 6 except for SAB where NF + V = 3, NF + HT = 5, HF + V = 6, HF + HT = 4). Asterisks denote statistical significance: * p<0.05 relative to NF + V in (E and F), indicated groups in (G). Scale bar measures 100 μm.

Unlike findings in the young animals, HFD did not affect significantly alter Aβ burden in early middle-aged female 3xTg-AD mice. HT resulted in a significant decrease in Aβ accumulation in mice maintained on normal diet, lowering Aβ load by 46% in subiculum and 68% in CA1 (Fig 4A, 4B and 4E; two-way ANOVA; subiculum, F_hormone_(1,21) = 9.76, p<0.05; Newman-Keuls test: NF + V vs NF + HT p<0.05; CA1, F_hormone_(1,21) = 16.71, p<0.005; Newman-Keuls test: NF + V vs NF + HT p<0.005; NF + V vs HF + HT p<0.005). Interestingly, the Aβ-lowering effects of HT were diminished in HFD mice, yielding a nonsignificant 26% decrease of Aβ in the subiculum and 43% in hippocampus CA1 (Fig 4C–4E). Phosphorylated tau staining (S2 Fig) showed a non-significant trend of decreased levels with HFD (Fig 4F; two-way ANOVA; F_diet_(1,22) = 3.07, p = 0.09) and a significant decrease by HT (Fig 4F; two-way ANOVA; F_hormone_(1,22) = 4.91, p<0.05). HT-treated mice in both the normal and HFD groups showed significantly better performance on SAB compared to vehicle-treated mice (Fig 4G; two-way ANOVA; F_hormone_(1,16) = 18.81, p<0.005; Newman-Keuls test NF + V vs NF + HT p<0.05; HF + V vs HF + HT p<0.05).

### Effects of hormone therapy on metabolic and AD-related outcomes in late middle-aged mice

**Fig 5:**
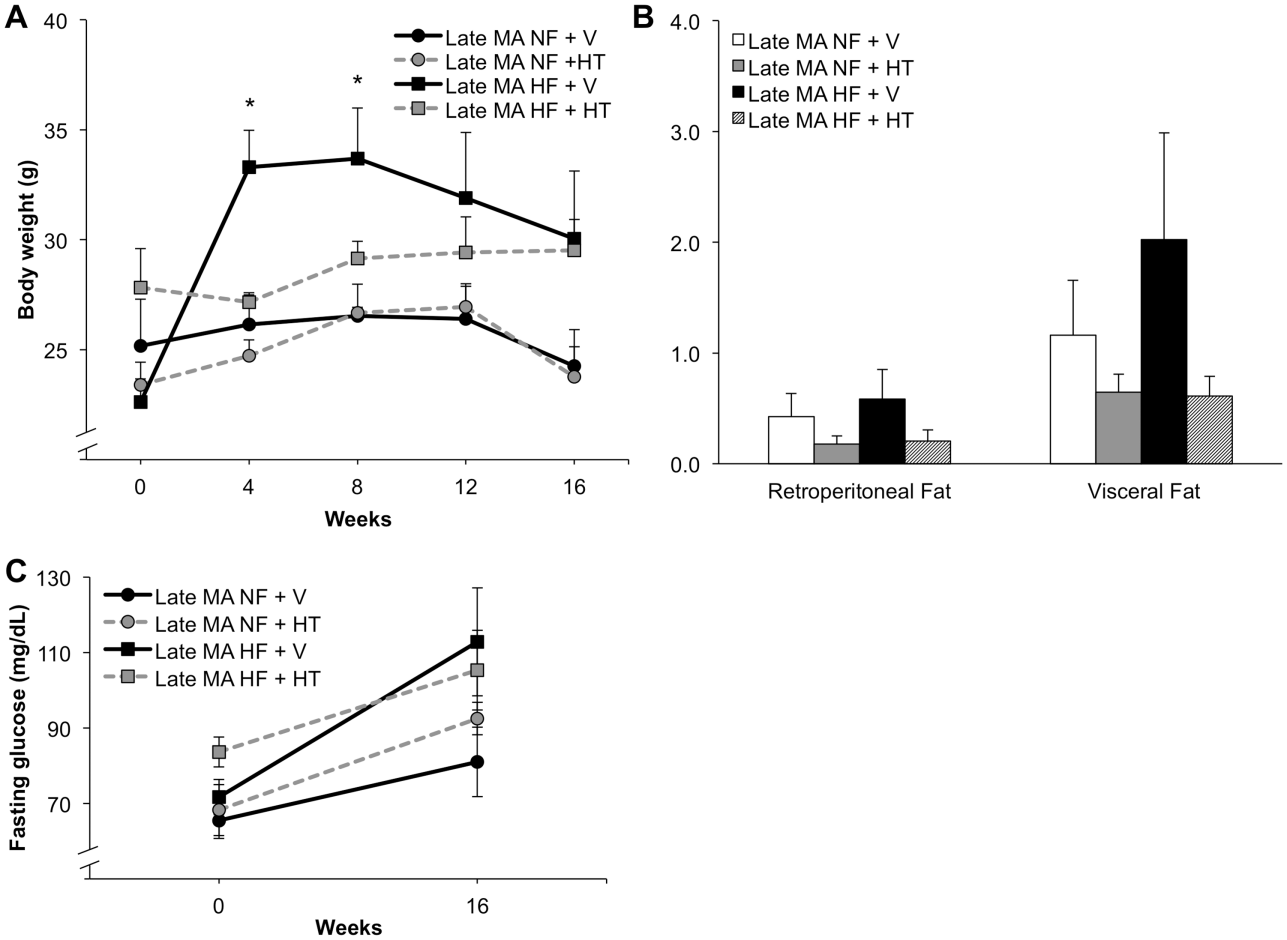
Effects of hormone therapy and high-fat diet on metabolic outcomes in late middle-aged female mice. (A) Late middle-aged (MA, 16 month old) female mice trended toward protection from HF diet induced weight gain when treated with HT, but only the diet-induced weight gain showed statistical significance. (B) Relative fat pad weights showed non-significant trends of increased mass with HF diet and decreased mass by HT in late middle-aged mice. (C) No significant differences in fasting glucose levels were observed in HT-treated late middle-aged females. In (A) and (C) black circles with a solid line denote NF + V animals, grey circles with a dashed line denotes NF + HT, black squares with a solid line denote HF + V animals, and grey squares with a dashed line denotes HF + HT. In (B), white bar indicates NF + V, grey indicates NF + HT, black indicates HF + V, and striped indicates HF + HT. Data show mean ± SEM (NF + V = 6, NF + HT = 4, HF + V = 6, HF + HT = 6). Asterisks denote statistical significance: * p<0.05 relative to NF + V group.

In late middle-aged, reproductively senescent animals (age 16–17 mo at start of treatment), HFD was associated with significant weight gain only at weeks 4 and 8 (Fig 5A; two-way repeated measures ANOVA; F(12,72) = 3.53; p<0.001; Bonferoni test: NF + V vs HF + V at 4 and 8 weeks, p<0.05). All groups except HF + HT exhibited trends of reduced body weight over the last month at which time the mice were 19–20 months of age, a period marked by declining health and increasing frailty and mortality in this strain [26]. Fat pad weights showed modest, statistically non-significant trends of being increased by HFD and reduced by HT (Fig 5B; two-way ANOVA; retroperitoneal, F(1,18) = 0.10, p = 0.75; visceral, F(1,18) = 0.57, p = 0.46). Neither HFD nor HT significantly affected fasting glucose levels (Fig 5C; two-way repeated measures ANOVA; F (3,19) = 2.28, p = 0.11). Finally, plasma levels of cholesterol but not triglycerides were significantly increased by HFD (Table 1).

**Fig 6:**
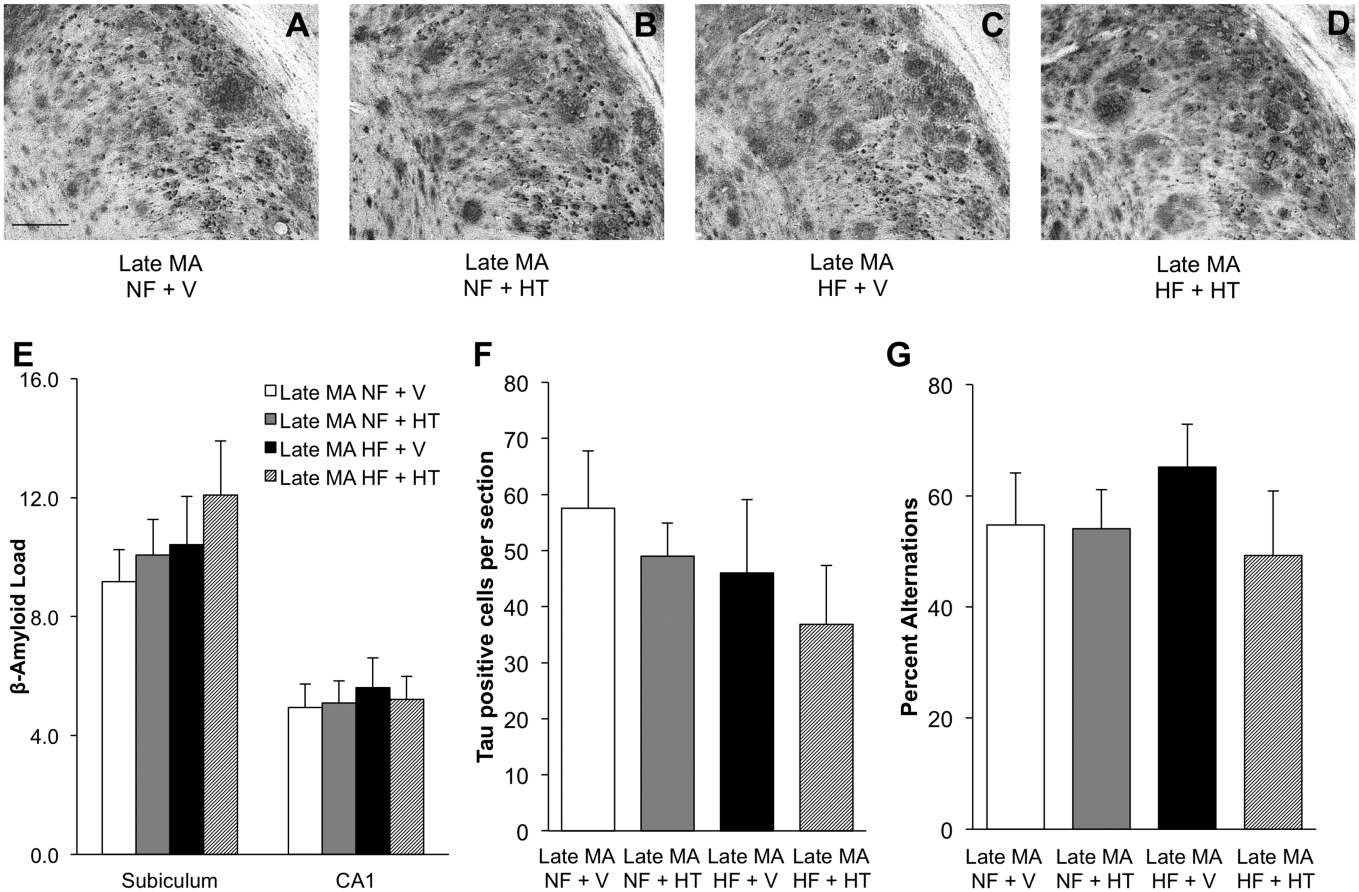
Effects of hormone therapy and high-fat diet on amyloid load and cognitive performance in late middle-aged female mice. Representative pictures of β-amyloid immunoreactivity in the subiculum of late middle-aged (MA) mice demonstrate that neither HT nor HF diet significantly affected β-amyloid accumulation: (A) late middle-aged NF + V, (B) late middle-aged NF + HT, (C) late middle-aged HF + V, and (D) late middle-aged HF + HT. (E) Quantification of β-amyloid load showed that there was no effect of treatments in late middle-aged female mice. (F) The levels of positive tau staining were not affected by either diet or HT in the late middle-aged mice. (G) Similarly, performance on the spontaneous alternation task showed no significant differences across groups. In (E-G), white bar indicates NF + V, grey indicates NF + HT, black indicates HF + V, and striped indicates HF + HT. Data show mean ± SEM (NF + V = 6, NF + HT = 4, HF + V = 6, HF + HT = 6, except for SAB where NF + V = 4, NF + HT = 4, HF + V = 6, HF + HT = 4). Scale bar measure 100 μm.

HFD did not alter Aβ load in the older mice. Unlike observations in the early middle-aged mice, HT was ineffective in reducing Aβ burden in subiculum and hippocampus CA1 of late middle-age animals regardless of diet (Fig 6A–6E; two-way ANOVA; subiculum, F_hormone_(1,17) = 0.72, p = 0.41; CA1, F_hormone_(1,17) = 0.02, p = 0.89). Neither the levels of phosphorylated tau (Fig 6F; two-way ANOVA; F_hormone_(1,17) = 0.83, p = .37) nor performance on the spontaneous alternation test (Fig 6G; two-way ANOVA; F_hormone_(1,15) = 0.82, p = 0.38) significantly differed across the groups.

### Comparison of outcomes across age groups

While the primary goal of this study is to understand how the effects of diet-induced obesity and its modulation by HT differ with aging, the findings also provide some insight into effects of diet and aging on outcome measures irrespective of HT. Comparison of values across young adult, early middle-age and late middle-age mice on control versus HFD shows simple main effects of diet on body weight, retroperitoneal and visceral fat pad weight, and fasting glucose (Table 2). Similarly, aging was associated with significant main effects on body weight, visceral fat pad weight, Aβ load, and microglial activation (Table 2). Significant interactions of diet and age were observed only for retroperitoneal fat pad weight and microglial activation (Table 2).

**Table 2:** Independent and interactive effects of age and diet on outcome measures in young, early middle-age and late middle-age mice.

Measure	Diet	Aging	Interaction
Body weight	F(1,35) = 30.39, p< 0.05	F(2,35) = 3.72, p< 0.05	F(2,35) = 1.85, p = 0.17
Retroperitoneal fat	F(1,34) = 30.89, p< 0.05	F(2,34) = 3.26, p = 0.051	F(2,34) = 5.10, p< 0.05
Visceral fat	F(1,34) = 26.59, p< 0.05	F(2,34) = 3.64, p< 0.05	F(2,34) = 2.78, p = 0.08
Fasting glucose	F(1,35) = 15.18, p< 0.05	F(2,35) = 1.49, p = 0.24	F(2,35) = 1.19, p = 0.32
β-Amyloid load	F(1,28) = 0.82, p = 0.37	F(2,28) = 5.73, p< 0.05	F(2,28) = 0.78, p = 0.47
Spontaneous alternation	F(1,24) = 0.19, p = 0.67	F(2,24) = 1.56, p = 0.23	F(2,24) = 2.36, p = 0.11
Microglial activation	F(1,34) = 3.93, p = 0.06	F(2,34) = 101.6, p< 0.05	F(2,34) = 4.44, p< 0.05

### Effects of aging, high-fat diet, and hormone therapy on microglial activation

**Fig 7:**
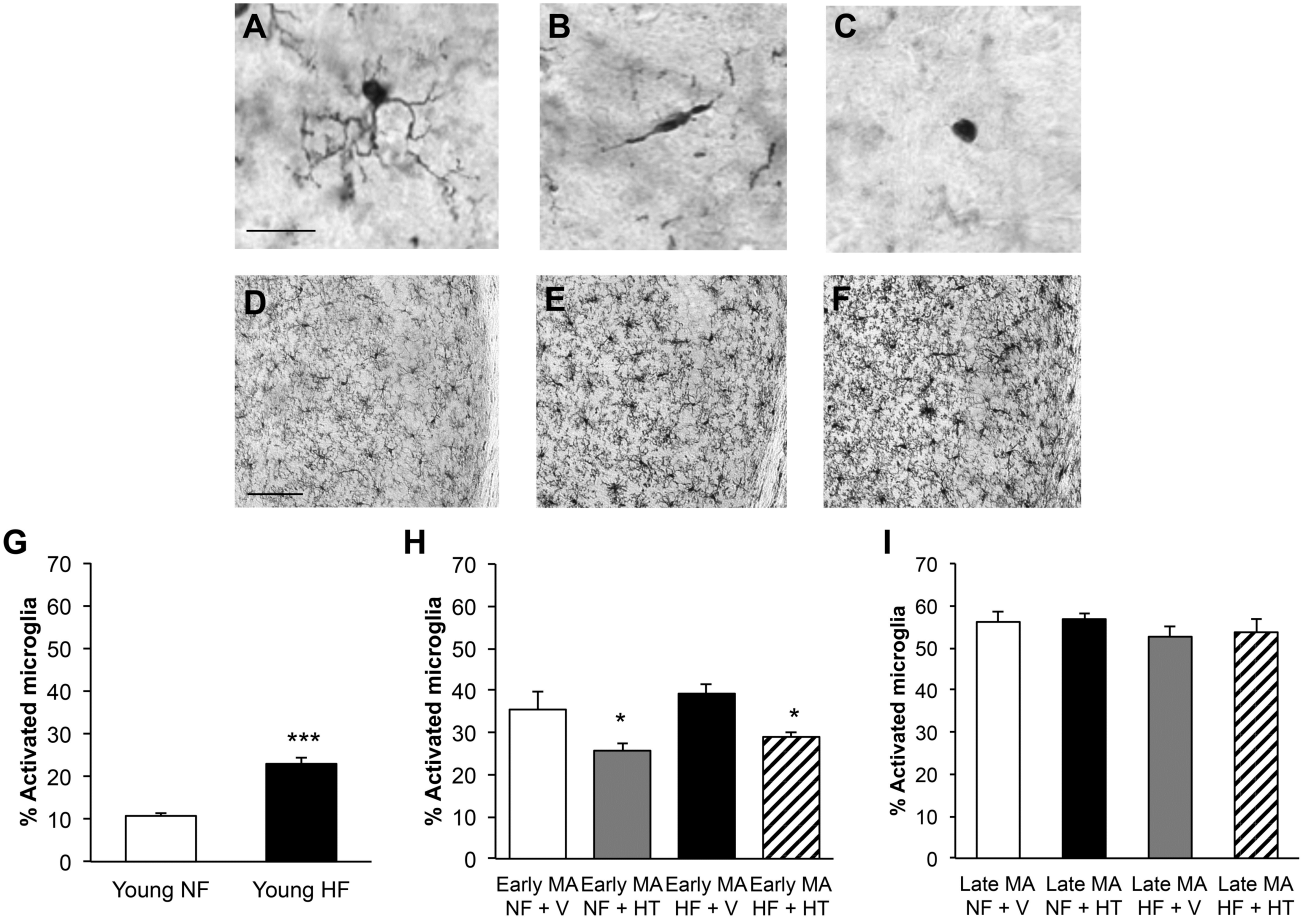
Effects of hormone therapy and high-fat diet on microglia in female mice. Microglia were morphologically classified into three types: (A) Type 1 are highly ramified and were considered non-activated; (B) Type 2 have rod-shaped cell bodies and only short, mostly unbranched processes; (C) Type 3 have round cell bodies with no processes or only filopodia. The number of microglia in female 3xTg-AD mice visually increased with aging even without treatment: (D) young NF, (E) early middle-aged NF + V and (F) late middle-aged NF + V. (G) The percentage of activated microglia (types 2 and 3) increased with HF diet treatment in young females. HT was able to decrease the percentage of activated microglia in (H) early middle-aged but not (I) late middle-aged mice. In (G) white indicates Young NF (n = 6) and black indicates Young HF (n = 7). In (H), white bar indicates NF + V (n = 7), grey indicates NF + HT (n = 5), black indicates HF + V (n = 8), and striped indicates HF + HT (n = 6). In (I), white bar indicates NF + V (n = 6), grey indicates NF + HT (n = 4), black indicates HF + V (n = 6), and striped indicates HF + HT (n = 6). Data show mean ± SEM. Asterisks denote statistical significance: *** p<0.0001 relative to Young NF in (G), * = p<0.05 relative to NF+ V and HF + V, respectively in (H). Scale bar in (A) measure 20 μm and in (D) measures 100 μm.

Activated microglia are observed in obesity and AD and may contribute to the progression of both conditions. We evaluated the number and activation state of microglia across ages and treatment conditions using morphological analysis of Iba-1 staining. Type 1 or resting microglia exhibit circular cell bodies with thin, highly ramified processes (Fig 7A). Type 2 and 3 microglia are considered activated by morphological criteria, with type 2 microglia showing rod-shaped somas with fewer thicker processes (Fig 7B) and type 3 microglia having a more amoeboid appearance (Fig 7C). There was a significant increase in the number of microglia per section in the hippocampus with increasing age (Fig 7D and 7E; Young NF: 37.1 +/- 2.7; Early Middle-Age NF + V: 60.7 +/- 5.1; Late Middle-Age NF + V: 87.3 +/- 9.4; one-way ANOVA; F(2,19) = 17.0, p<0.0001; Newman-Keuls test: Young NF vs Early Middle-Age NF, p<0.05; Young NF vs Late Middle-Age NF, p<0.0001; Early Middle-Age NF vs Late Middle-Age NF, p<0.005). In young animals, the percentage of activated microglia (types 2 and 3) increased with HFD (Fig 7F; t-test; t = 8.69, df = 11, p<0.0001). In early middle-aged females, the level of microglial activation was not significantly affected by HFD but was significantly reduced by HT in the both the control and HFD groups (Fig 7G; two-way ANOVA; F_hormone_(1,22) = 12.1, p<0.005; Newman-Keuls test: NF + V vs NF + HT, p<0.05; HF + V vs HF + HT, p<0.05). Neither HFD nor HT was associated with significantly altered microglial activation in late middle-aged mice (Fig 7H; two-way ANOVA; F_hormone_(1,18) = 0.11, p = 0.74).

### PCR analysis of inflammation and glial activation

In order to look more closely at microglial activation and neuroinflammation, RNA transcript levels for microglial activity markers (TSPO, CD11b, and CD33) and several cytokines (IL-1β, TNFα, and IL-6) were assessed (Table 3). All of the microglial markers showed significantly increased expression with age. In the early middle-age animals, HT groups showed lower CD33 expression and a trend (p = 0.07) towards reduced TSPO. No significant changes in microglial markers were observed across groups in the late middle-age animals. Among the cytokines, aging was associated with significantly increased levels of IL-1β and a trend (p = 0.051) towards elevated IL-6. IL-1β was the only cytokine increased by HFD in the young animals. Also, IL-1β was decreased by HT in early middle-age mice. No significant effects on cytokine expression were seen across groups in late middle-age.

**Table 3:** Relative levels of mRNA expression across treatment groups.

Gene	Young		Early MA		Late MA		Aging p value
	p value	Fold change	p value	Fold change	p value	Fold change	
TSPO	Diet = 0.61	NF = 1.00 ± 0.01	Diet = 0.57	NF + V = 1.09 ± 0.22	Diet = 0.14	NF + V = 2.72 ± 0.35	**< 0.0001**
				NF + HT = 0.73 ± 0.16		NF + HT = 2.17 ± 0.54	
		HF = 0.89 ± 0.24	*HT = 0*.*072*	HF + V = 0.98 ± 0.21	HT = 0.23	HF + V = 2.03 ± 0.39	
				HF + HT = 0.62 ± 0.11		HF + HT = 1.58 ± 0.38	
CD11b	*Diet = 0*.*052*	NF = 1.00 ± 0.02	Diet = 0.39	NF + V = 1.94 ± 0.30	Diet = 0.57	NF + V = 2.00 ± 0.30	**0.010**
				NF + HT = 2.14 ± 0.53		NF + HT = 2.50 ± 0.48	
		HF = 1.45 ± 0.22	HT = 0.60	HF + V = 1.74 ± 0.16	HT = 0.12	HF + V = 1.76 ± 0.19	
				HF + HT = 1.84 ± 0.19		HF + HT = 2.35 ± 0.38	
CD33	Diet = 0.43	NF = 1.01 ± 0.04	**Diet = 0.039**	NF + V = 1.87 ± 0.24	Diet = 0.87	NF + V = 2.47 ± 0.39	**0.0029**
				NF + HT = 1.46 ± 0.17		NF + HT = 2.03 ± 0.40	
		HF = 0.88 ± 0.16	**HT = 0.019**	HF + V = 1.53 ±0.18	HT = 0.60	HF + V = 2.34 ± 0.45	
				HF + HT = 0.91 ± 0.13		HF + HT = 2.31 ± 0.38	
IL-1β	**Diet = 0.001**	NF = 1.01 ± 0.05	*Diet = 0*.*10*	NF + V = 2.30 ±0.42	Diet = 0.58	NF + V = 3.44 ± 0.56	**0.002**
				NF + HT = 1.26 ± 0.29		NF + HT = 2.76 ± 0.58	
		HF = 0.69 ± 0.05	**HT = 0.027**	HF + V = 1.48 ± 0.21	HT = 0.67	HF + V = 2.69 ± 0.54	
				HF + HT = 1.07 ± 0.10		HF + HT = 2.84 ± 0.54	
TNFα	Diet = 0.93	NF = 1.02 ± 0.14	**Diet = 0.013**	NF + V = 1.60 ± 0.40	Diet = 0.88	NF + V = 1.95 ± 0.41	0.18
				NF + HT = 1.91 ± 0.53		NF + HT = 3.89 ± 1.19	
		HF = 1.05 ± 0.28	HT = 0.89	HF + V = 1.00 ± 0.32	*HT = 0*.*10*	HF + V = 2.74 ± 0.70	
				HF + HT = 0.60 ± 0.12		HF + HT = 3.34 ± 0.73	
IL-6	Diet = 0.42	NF = 1.01 ± 0.04	Diet = 0.61	NF + V = 1.89 ± 0.41	Diet = 0.59	NF + V = 1.89 ± 0.20	*0*.*051*
				NF + HT = 2.09 ±0.29		NF + HT = 2.39 ± 0.56	
		HF = 1.17 ± 0.20	HT = 0.91	HF + V = 1.88 ±0.31	HT = 0.74	HF + V = 2.48 ± 0.23	
				HF + HT = 1.75 ±0.19		HF + HT = 2.25 ± 0.53	

p < 0.05 denoted by bold numbers, p ≤ 0.10 denoted by italicized numbers

## Discussion

Previous work has identified mid-life increases in central obesity [42, 43] and decreases in ovarian hormones [44, 45] as risk factors for the development of AD. Since estrogens are negative regulators of obesity and its associated metabolic disturbances [46, 47], estrogen-based HT may be expected to improve both AD- and obesity-related outcomes. However, findings in human [13, 48] and rodent [49] studies suggest that the neural benefits of estrogens may be limited to early phases of reproductive senescence. To investigate these issues, we evaluated the individual and interactive effects of estrogen-based HT and diet-induced obesity on metabolic and AD-related measures in female 3xTg-AD mice at increasing ages. Our findings show that the deleterious effects of diet-induced obesity and beneficial actions of HT are dependent upon age-related changes in AD-related pathology and reproductive senescence, respectively.

Our results suggest that diet-induced obesity significantly accelerates only the initial development of AD-related pathology in AD transgenic mice. In the 3xTg-AD mouse model of AD, intraneuronal and neuropil accumulation of Aβ are not apparent until several months of age, with robust pathology and cognitive deficits arising in early middle-age [27, 30, 50–53]. It is important to note that our method of quantifying Aβ has been optimized for 3xTg-AD such that it detects Aβ but neither amyloid precursor protein nor its carboxyl-terminal fragments, both of which exhibit qualitatively distinct temporal and staining characteristics [30]. Here we observed that HFD significantly exacerbated Aβ burden and behavioral impairment when introduced in early life, prior to significant preexisting pathology. This finding is consistent with our previous observation that four-month exposure to HFD introduced at age 3 mo significantly increased Aβ load and worsened performance in the spontaneous alternation task in male and female 3xTg-AD mice [38]. Similar findings of worsened AD-related pathology by early exposure to high-fat and/or high sugar diets have been reported in other AD transgenic strains, including Tg2576 [54], APP*Swe/Ind* [55], and APP/PS1 [56]. However, we also found that HFD initiated in 3xTg-AD mice at early and late middle-ages, periods when pathology is already well-established in 3xTg-AD mice, did not further increase Aβ burden, microglial activation, or behavioral impairment. This absence of significant exacerbation of AD-related pathology by HFD is generally consistent with recent observations that HFD fails to significantly increase Aβ burden and phospho-tau positive neurons in male 3xTg-AD at similar early and late middle-ages [57]. Together, these findings suggest that obesity may foster early AD pathogenesis but not significantly drive subsequent disease progression. This position is consistent with the human literature [58], which generally indicates that obesity is a risk factor for development of AD at midlife [42, 43] but less so in late-life [59]. If obesity is a factor for the development rather than the progression of AD neuropathology, then interventions to prevent and or reduce obesity should be directed towards young adult and middle-aged people.

A second key finding from this study is that estrogen-based HT induces neuroprotective effects in early but not late middle-age. Previous work from our group [30, 36, 60] and others [61–63] has established that estradiol attenuates AD-related pathology in young adult female rodents. The extent to which estradiol protects against AD-related neuropathology across naturally occurring reproductive aging in AD models has not been evaluated. We observed that initiation of HT at 7–9 mo significantly reduced Aβ burden, microglial activation, and HFD-induced obesity while also improving behavioral performance in female 3xTg-AD mice. Interestingly, the effects of HT on Aβ burden were attenuated in obese mice, suggesting that the effects of obesity in early middle-age may impair protective estradiol-mediated actions and/or accelerate their age-related loss. Numerous neural benefits of estradiol have been assessed in the context of chronological and reproductive aging, with many actions diminished with advancing age [64, 65] but others largely spared [66, 67]. In contrast to the beneficial effects of HT in early middle-age, we found that HT initiated at late middle-age (16–17 mo) yielded no improvement in behavior and no significant reduction in microglial activation or Aβ burden. A recent study that assessed only aged 3xTg-AD mice found a similar absence of estradiol benefit [68]. These observations provide compelling support for the critical window hypothesis, which posits that HT is effective at delaying and/or preventing dementia when initiated in early middle-age women near the onset of menopause but is without benefit and perhaps harmful in late middle-aged and aged women [13, 19].

Accumulating evidence suggests that ovarian hormone depletion and increasing adiposity at midlife are independent and perhaps cooperative risk factors for the development of AD in women [69]. How HT affects these risk factors and their interactions during aging remains to be defined. Prior work found that estradiol retains its ability to reduce body weight in aged rats [70]. We found that although the neuroprotective effects of HT were absent in late middle-age, the metabolic effects of HT were apparent though reduced in magnitude. Animals on HFD receiving vehicle weighed more and trended towards larger fat depots than those receiving a normal fat diet, even in the late middle-aged group. We observed significant HFD-induced increases in plasma levels of total cholesterol but not triglycerides. Although HFD in rodents is typically associated with elevations in both cholesterol and triglycerides [71], many studies find no significant changes and sometimes even decreased levels in plasma triglycerides [72] in an apparently strain-specific manner [73, 74]. HT was associated with reduced body weight in both early and late middle-aged mice. These findings are generally consistent with some of the human data, which suggest that several metabolic outcomes are improved by HT [11]. Interestingly, the benefits of HT on obesity-related measures paired with the attenuation of HT-mediated Aβ reduction in obese mice collectively suggest that regulation of metabolism may not be the key mechanism linking ovarian hormones, obesity, and AD.

Microglial activation and its regulation of neuroinflammation are implicated as key components of AD pathogenesis [8], the neural consequences of obesity [75, 76], and the interactions between obesity and AD [69]. Although some microglia-mediated actions, including phagocytosis, may combat aspects of AD neuropathology [77], chronic microglial activation is thought to precede and promote AD pathogenesis [78–80]. HFD has been shown to activate microglia and contribute to AD progression [81, 82]. Consistent with this, we observed that HFD increased both a morphological marker of microglial activation and Aβ accumulation in young female 3xTg-AD mice, but interestingly did not significantly affect either outcome in early and late-middle aged animals. A similar effect has been seen with long-term HFD in 3xTg-AD males, in which microglial activation was increased in younger but not aged mice maintained on HFD [57]. The association seen here suggests a potential causal linkage between HFD, microglial activation, and development of pathology, although in other studies no correlation was observed [57]. Interestingly, we did not observe increases in expression of pro-inflammatory cytokines with HFD. Although diet-induced obesity increases features of neuroinflammation including glial activation [83], obesogenic diets often do not yield increased cytokine expression [84] or do so in a brain-region specific manner [85] even with evidence of significant microglial activation [86]. This disconnection between microglial activation and cytokine expression may be especially prevalent in female mice, which are relatively resistant to HFD [38, 87]. Thus, features of neuroinflammation beyond a generalized increase in cytokine expression may underlie the link between microglial activation and HFD-induced acceleration of AD-like pathology. In the 3xTg-AD model, interventions that reduce microglial activation and its associated neuroinflammation also reduce pathology [88–90]. Estrogens, and to some extent progesterone, exert significant anti-inflammatory effects in multiple tissues [91] including the brain [92, 93]. We found that the efficacy of HT in reducing Aβ burden and improving behavior was predicted by its ability to reduce indices of microglial activation. While additional studies will be required to rigorously define the relationship, the findings suggest that obesity-induced activation of microglia contributes to AD-related pathology and that HT can attenuate both outcomes in early but not late middle-age.

There are some limitations to this study. First, there was significant mortality in the late middle-age mice, particularly in the HT-treated groups. Concerns of low animal number are mitigated to some extent by excess animal enrollment. However, there remains the possibility that surviving mice are not representative in terms of how aging mice respond to HFD and HT. The cause of mortality is uncertain, though deceased middle-aged mice typically exhibited evidence of pyometra. In rodents, estradiol treatment [94], particularly when delivered chronically [95, 96], is associated with development of urinary tract infections including pyometra. Further, 3xTg-AD mice exhibit accelerated immunosenescence [97] and mortality [26], which may have interacted with HT to yield poor health outcomes in aging female 3xTg-AD mice. Second, we did not consider potential differences in circulating estradiol levels associated with adiposity, which was increased by both aging and HFD. Adipose tissue can promote increased generation of estradiol and other steroid hormones [98]. Note that the high levels of estradiol achieved with our HT protocol are greater than those associated with obesity. Third, our quantitative PCR analyses of microglia markers and cytokines prevented more detailed biochemical assessments of Aβ and tau. The immunohistochemical analyses are certainly valid but are not indicative of the subtler differences that may occur across conditions, for example changes in relative levels of soluble, oligomeric and detergent-insoluble Aβ fractions. Finally, as with most AD transgenic models, there are inherent difficulties with 3xTg-AD mice in terms of defining interactions with aging since neuropathology begins prior to middle-age. Thus, our observation that diet-induced obesity increases pathology in young adult but not in early and late middle-age mice may reasonably be associated with either chronological age or the level of neuropathology. Additional research will be required to pursue these issues.

## Conclusion

In summary, we have provided novel findings on the individual and interactive effects of aging, obesity, and estrogen-based HT on the development of AD-related pathology. First, our results demonstrate that diet-induced obesity promotes the initial phases of AD neuropathology but has no effect on the progression of established pathology. Second, we provide the first definitive evidence in an AD model that directly supports the critical window hypothesis, which posits that HT significantly delays the onset and/or reduces the risk of AD only when initiated near the onset of menopause when the brain retains high estrogen responsiveness. Third, we observe important interactions between obesity and HT. Although HT attenuates some effects of diet-induced obesity even in aged mice, we found that obesity significantly diminished the neuroprotective effects of HT in middle-aged mice. Finally, our data are consistent with a role of microglia as a significant mediator of the observed effects, with morphological and molecular evidences of microglial activation predicting the deleterious effects of obesity and the protective effects of HT on AD-related pathology. Collectively, these findings suggest that the perimenopause is an important transition that affects the independent and interactive effects of obesity and HT on development of AD in women.

## Supporting information

S1 Fig Immunoreactivity levels of Aβ but not C-terminal fragments (CTF) of amyloid precursor protein increase with age in 3xTg-AD females. (A-C) CTF immunoreactivity does not change with age. (D) A high magnification (100x) of CTF staining in subiculum shows immunoreactivity is largely in cell periphery and/or membranes of neurons. (E-G) Aβ staining increases with age. Plaques become visible at middle-age (MA) with many more apparent in late middle-age mice. (H) A high magnification image of Aβ staining in subiculum shows both diffuse and punctate intraneuronal staining that qualitatively differs from the pattern of CTF immunostaining. Scale bar measures 100 μm in (A) and 20 μm in (D).
(PDF)

S2 Fig Phospho-tau immunoreactivity increases with age in female 3xTg-AD mice. Images show AT8 phospho-tau immunostaining from early middle-aged (MA) mice maintained on (A) normal and (C) high-fat diets. Increased numbers of AT8-immunreactive cells in late middle-ages mice under both dietary conditions (B, D). Scale bar measures 50 μm.
(PDF)
