# Supplementary figures and images for: Age-dependent regulation of obesity and Alzheimer-related outcomes by hormone therapy in female 3xTg-AD mice
Source: PLoS One. 2017 Jun 2;12(6):e0178490. doi: 10.1371/journal.pone.0178490 (PMC5456100; doi:10.1371/journal.pone.0178490)

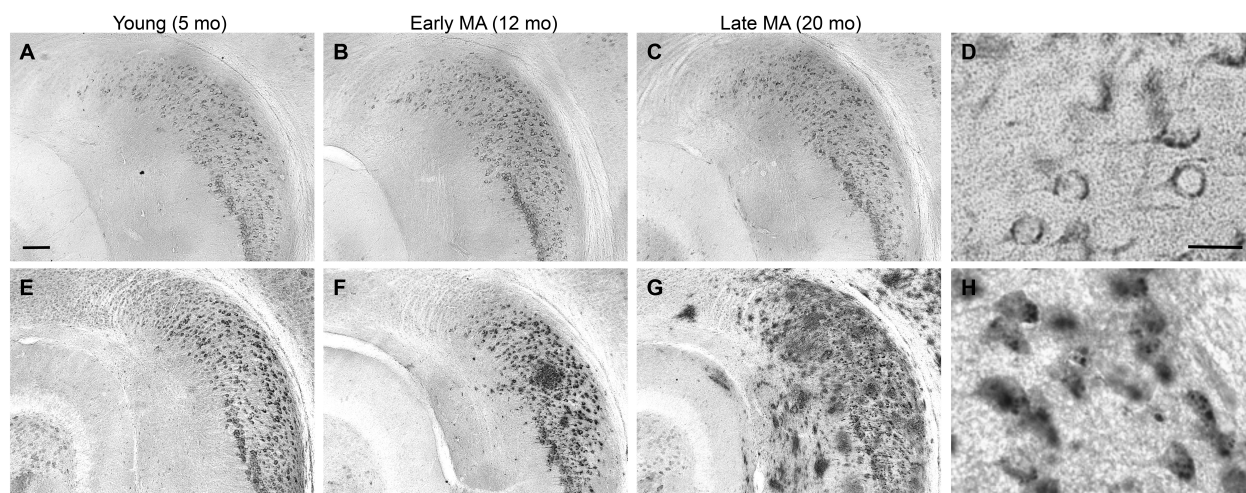

Supplement: S1 Fig — (A-C) CTF immunoreactivity does not change with age. (D) A high magnification (100x) of CTF staining in subiculum shows immunoreactivity is largely in cell periphery and/or membranes of neurons. (E-G) Aβ staining increases with age. Plaques become visible at middle-age (MA) with many more apparent in late middle-age mice. (H) A high magnification image of Aβ staining in subiculum shows both diffuse and punctate intraneuronal staining that qualitatively differs from the pattern of CTF immunostaining. Scale bar measures 100 μm in (A) and 20 μm in (D). (PDF) [file pone.0178490.s001.pdf]

Early MA (12 mo)

Late MA (20 mo)

Normal Fat

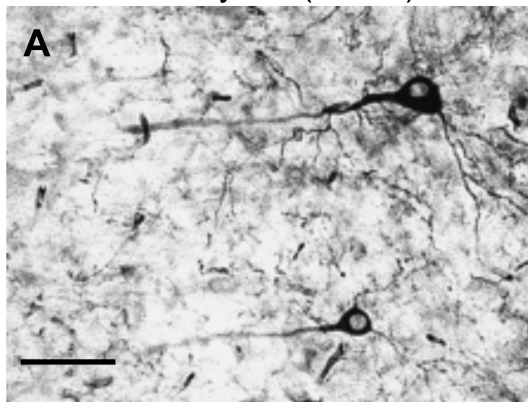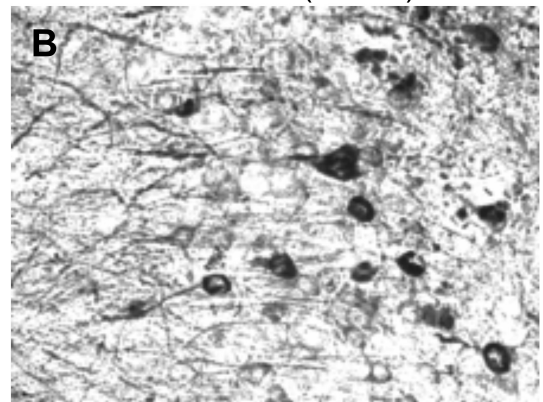

High Fat

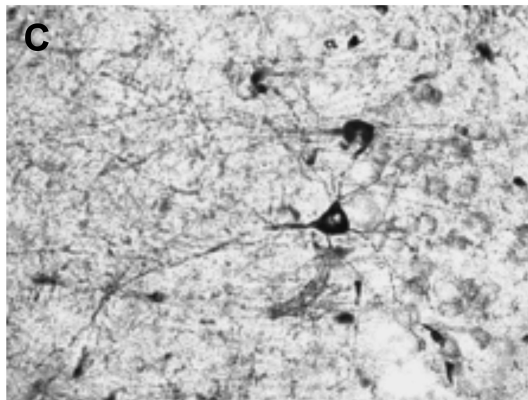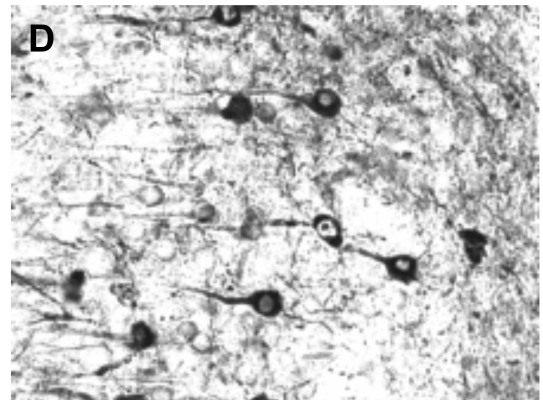

Supplement: S2 Fig — Images show AT8 phospho-tau immunostaining from early middle-aged (MA) mice maintained on (A) normal and (C) high-fat diets. Increased numbers of AT8-immunreactive cells in late middle-ages mice under both dietary conditions (B, D). Scale bar measures 50 μm. (PDF) [file pone.0178490.s002.pdf]
